# Distinct Metabolic features differentiating FLT3-ITD AML from FLT3-WT childhood Acute Myeloid Leukemia

**DOI:** 10.1038/s41598-018-23863-9

**Published:** 2018-04-03

**Authors:** Bradley Stockard, Timothy Garrett, Joy Guingab-Cagmat, Soheil Meshinchi, Jatinder Lamba

**Affiliations:** 1 Department of Pharmacotherapy and Translational Research, College of Pharmacy, University of Florida, Gainesville, FL USA; 2 Southeast Center for Integrated Metabolomics, University of Florida, Gainesville, FL USA; 3 Clinical Research Division, Fred Hutchinson Cancer Research Center, Seattle, WA USA; 4 Department of Medicine/Division of Hematology, University of Washington, Seattle, WA USA

## Abstract

Acute myeloid leukemia (AML) is a heterogeneous disease with dismal response warranting the need for enhancing our understanding of AML biology. One prognostic feature associated with inferior response is the presence of activating mutations in FMS-like tyrosine kinase 3 (FLT3) especially occurrence of internal tandem duplication (FLT3-ITD). Although poorly understood, differential metabolic and signaling pathways associated with FLT3-ITD might contribute towards the observed poor prognosis. We performed a non-targeted global metabolic profiling of matched cell and plasma samples obtained at diagnosis to establish metabolic differences within FLT3-ITD and FLT3-WT pediatric AML. Metabolomic profiling by Ultra-High Performance-Liquid-Chromatography–Mass Spectrometry identified differential abundance of 21 known metabolites in plasma and 33 known metabolites in leukemic cells by FLT3 status. These metabolic features mapped to pathways of significant biological importance. Of interest were metabolites with roles in cancer, cell progression and involvement in purine metabolism and biosynthesis, cysteine/methionine metabolism, tryptophan metabolism, carnitine mediated fatty acid oxidation, and lysophospholipid metabolism. Although validation in a larger cohort is required, our results for the first time investigated global metabolic profile in FLT3-ITD AML.

## Introduction

Acute myeloid leukemia (AML) is a heterogeneous disease characterized by complex molecular and cytogenetic abnormalities. These abnormalities are used for risk stratification and prediction of clinical outcome in AML. In addition to cytogenetic lesions, a number of mutations of prognostic significance have been identified using next generation sequencing efforts in recent years. One of the genes of prognostic significance in AML is fms-like tyrosine kinase 3 (FLT3). FLT3 plays role in the regulation of cell proliferation, differentiation and survival^1,2^. Mutations in FLT3, especially internal tandem duplication (ITD) in the juxta-membrane domain (FLT3-ITD), are present in around 20% of AML patients with predominant occurrence in patients with cytogenetically normal AML. The presence of FLT3-ITD mutation results in constitutive activation of FLT3 signaling and has been associated with poor prognosis and clinical outcome in response to chemotherapy^2–6^ Tyrosine kinase inhibitors such as midostaurin, sunitinib, sorafenib and ibrutinib have been investigated for treatment of FLT3-ITD positive AML^2,7^. However, development of drug resistance to FLT3-inhibitors has been a major challenge in successful treatment of patients^8,9^, warranting the need for further understanding the complexity and biology of FLT3-ITD in AML.

The emerging field of metabolomics focuses on quantitating metabolic features to better understand biochemical alterations associated with cancer. In recent years, metabolic alterations of significant relevance have been identified in several solid tumors^10,11^ and hematological malignancies such as chronic lymphocytic leukemia^12^, childhood acute lymphoblastic leukemia^13,14^ and adult AML^15,16^. A distinct glucose metabolism signature and 2–hydroxyglutarate levels, which is also referred to as an ‘oncometabolite’ have been reported to be of prognostic significance in adult AML patients^15,16^ but there are no reports in pediatric AML. Among pediatric patients, differential metabolome in pediatric acute lymphoblastic leukemia patients as compared to healthy controls has been reported^13^. Overall, these studies show that metabolomics can be successfully applied in leukemia studies to improve our understanding of disease characteristics and variance in clinical outcomes in AML. Unfortunately, the metabolome of pediatric AML has not been studied and there is currently a gap in our understanding of any metabolic features that are specific to FLT3-ITD positive AML. Identifying the FLT3-ITD specific metabolic profile might enhance our understanding of the poor outcome in these patients as well as give insight of disease biology and drug resistance mechanism. Thus, the current study was designed with the goal to establish metabolomic differences between FLT3 wild-type (WT) and FLT3-ITD childhood AML patients.

## Results

### Patient cohort and metabolite profiling

Our study included matched plasma samples and leukemic cells obtained at diagnosis from 16 pediatric AML patients (FLT3-ITD n = 8 and FLT3-WT, n = 8) enrolled in the AAML1031 clinical trial (NCT01371981). Table 1 summarizes the patient characteristics; overall median age of the patients was 13 years. Plasma samples and matched leukemic cells were obtained primarily from peripheral blood samples from patients at diagnosis before initiation of chemotherapy.

**Table 1:** Characteristics of pediatric patients with FLT3-ITD or FLT3-WT AML.

Characteristics	FLT3-WT	FLT3-ITD
Total	8	8
Age (years)		
Mean	13.71	12.41
Range	6.89–19.42	4.16–17.79
Sex		
Male	3	3
Female	5	5
Race		
White	6	5
Black	1	0
Other	1	3
Allelic Ratio		
Average	0	2.88
Range	0	0.8–13.35
Cytogenetics		
Normal	3	6
Abnormal	5	2

Untargeted global metabolomics profiling was performed in both positive and negative ionization using Ultra-High performance Liquid Chromatography – Mass Spectrometry (UHPLC-MS) at Southeast Center for Integrated Metabolomics (SECIM) University of Florida. A total of 2966 features were identified in plasma specimens and 1742 features in leukemic cells. Using our internal metabolite library that is regularly updated by running standards, we were able to annotate 290 metabolites in plasma and 143 metabolites identified in cells to known metabolites of which 58 were common between both types of specimens.

### Plasma Metabolic features differentiating FLT3-ITD and FLT3-WT AML

We utilized Metaboanalyst software^17^ to identify features separating FLT3-ITD from FLT3-WT AML. A total of 209 features were significantly different in abundance between FLT3-ITD and FLT3-WT groups at FDR <0.05 (volcano plot in Supplementary Figure 1 shows the separation of the two groups, Supplementary Table 1 provides list of metabolites). Among these 21 were annotated to known metabolites and were differentially abundant by FLT3 status as shown in Table 2. Metabolites with higher abundance in FLT3-ITD vs. FLT3-WT included: (1) organic acids: 3-methyl-2-oxovaleric acid associated with isoleucine metabolism, pyridine-2,3-dicarboxylate, 6-carboxyhexanoate (also commonly known as pimelic acid which has been reported to be higher in patients with uremic serum)^18^ and methyl indole-3-acetate; (2) amino acids and intermediates such as guanine, N-acetyl arginine, N-alpha-acetyl-L-Lysine, N-acetyl-DL-glutamic acid, L-carnitine, N-acetyl glycine, GABA, N-acetyl-amine, cysteine-S sulfate, and threonine/homoserine; (3) phosphocholine. Metabolites L-cysteic acid and asparagine were less abundant in FLT3-ITD vs. FLT3-WT patients.

**Table 2:** Metabolites with significantly differential abundance in plasma samples from patients with FLT3-ITD vs. without FLT3-ITD.

Metabolite	Classification	Associated Pathway	p-value	FDR	Fold Change
Guanine	Nucleosides	Purine metabolism/biosynthesis	1.09E-08	8.01E-06	5.722
Pyridine-2,3-Dicarboxylate	Organic Acids	Nicotinate and nicotinamide metabolism; beta-Alanine metabolism; tryptophan metabolism	8.75E-07	1.98E-04	3.6429
N-Alpha-Acetyl-L-Lysine	Amino Acids	Lysine synthesis	1.36E-06	2.43E-04	6.0508
N-Acetylglycine	Amino Acids	Arginine and proline metabolism	2.33E-06	3.42E-04	3.6015
GABA	Amino Acids	Alanine, aspartate and glutamate metabolism; Butanoate Metabolism; beta-Alanine Metabolism	3.74E-06	4.78E-04	2.7014
N-Acetyl-L-Alanine	Amino Acids	Arginine and proline metabolism	8.90E-06	8.09E-04	2.5869
Phosphocholine	Amines	Glycerophospholipid metabolism	2.74E-05	1.78E-03	1.8098
Diphenylamine	Xenobiotics	N/A	3.54E-05	2.17E-03	2.0411
3-Methyl-2-Oxovaleric Acid	Organic Acids	Isoleucine Metabolism	1.13E-04	4.83E-03	43.236
L-Carnitine	Amino Acids	Fatty Acid Metabolism	1.74E-04	6.16E-03	1.9491
Cysteine-S-sulfate	Amino Acids	Cysteine and methionine metabolism	2.27E-04	7.03E-03	3.7072
6-Carboxyhexanoate	Organic Acids		2.76E-04	8.20E-03	8.1077
Methyl Indole-3-Acetate	Organic Acids	Tryptophan metabolism	4.70E-04	0.01191	1.8294
Threonine/Homoserine	Amino Acids	Aminoacyl-tRNA biosynthesis; Cysteine and methionine metabolism	7.74E-04	0.017915	4.3347
N-Acetyl-DL-Glutamic Acid	Amino Acids	Urea Cycle	9.51E-04	0.019802	1.774
N-Acetyl-Arginine	Amino Acids	Protein/Amino Acid biosynthesis	1.27E-03	0.024796	11.915
*Asparagine*	*Amino Acids*	*Alanine, aspartate and glutamate metabolism; Nitrogen Metabolism; Cyanoamino acid Metabolism*	*1.60E-03*	*0.030212*	*0.4477*
*L-Cysteic Acid*	*Amino Acids*	*Taurine and hypotaurine metabolism; Cysteine and methionine metabolism*	*2.27E-03*	*0.038341*	*0.23168*
4-Acetamidobutanoate	Amino acids	N/A	2.27E-03	0.038341	1.9979
3-Hydroxydecanoic acid	Organic Acids	N/A	2.57E-03	0.041408	2.5453
Betaine	Amino acids	Glycine, serine, and threonine metabolism	2.59E-03	0.041654	1.6109

Note: Metabolites with lower abundance in FLT3-ITD are in italics.

**Figure 1:**
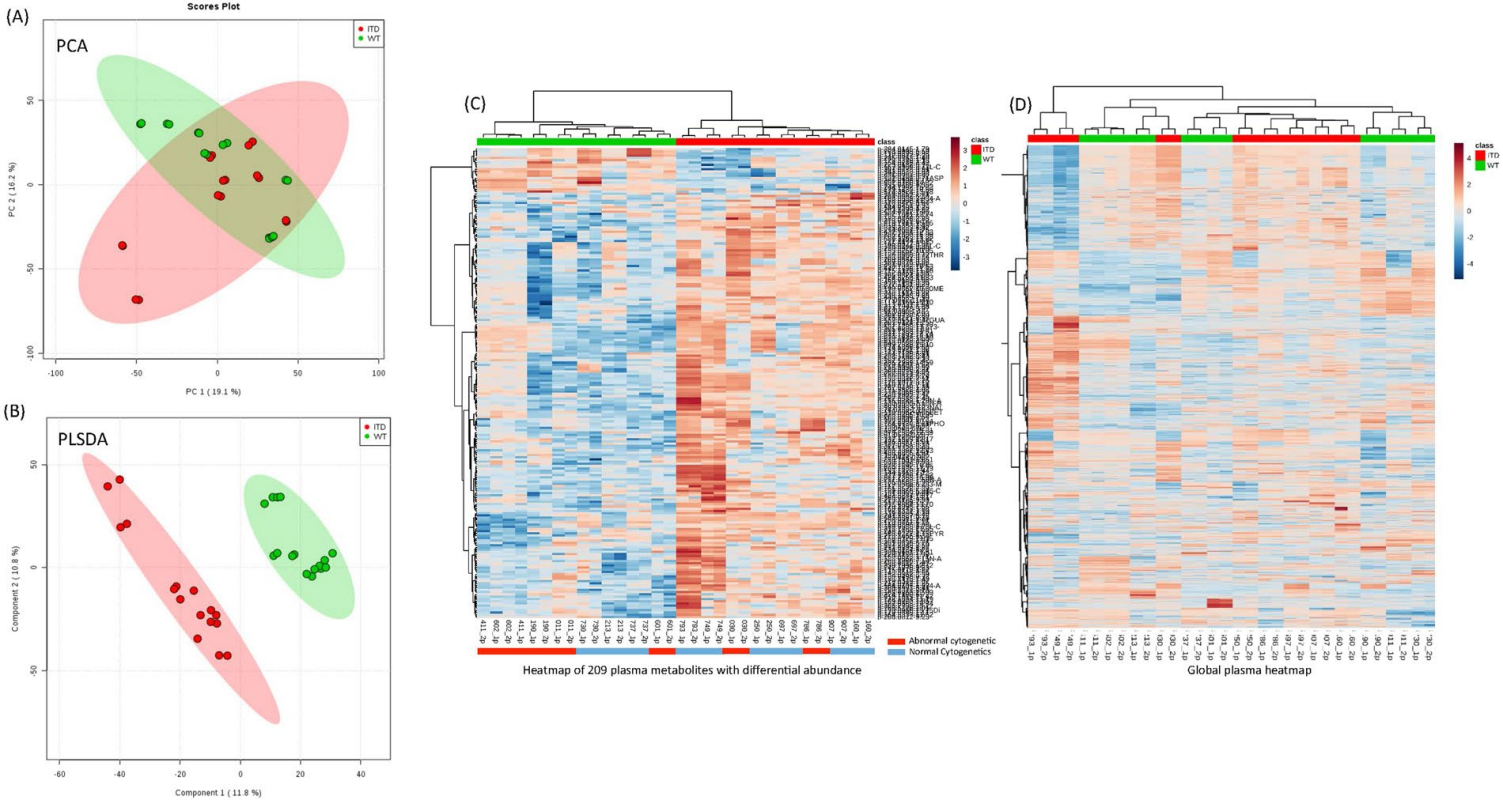
Plasma Metabolic differences between FLT3-ITD and FLT3-WT AML. (**A**) PCA plots of plasma samples. Unbiased multivariate analysis does not show clear separation of samples based on FLT3 status groups. (**B**) PLSDA plot of plasma samples shows global separation of pediatric AML patients by FLT3 status (FLT3-ITD n = 8 and FLT3-WT n = 8, all samples were run in duplicate). (**C**) Heatmap shows relative abundance patterns of 209 plasma metabolites (known and un-annotated) with significantly differential abundance according to FLT3 status. Clustering within the heatmap shows a clear distinction of several metabolites between in FLT-WT and FLT3-ITD patients. (**D**) Heatmap of the global metabolome for patient plasma samples from AML patients.

**Figure 2:**
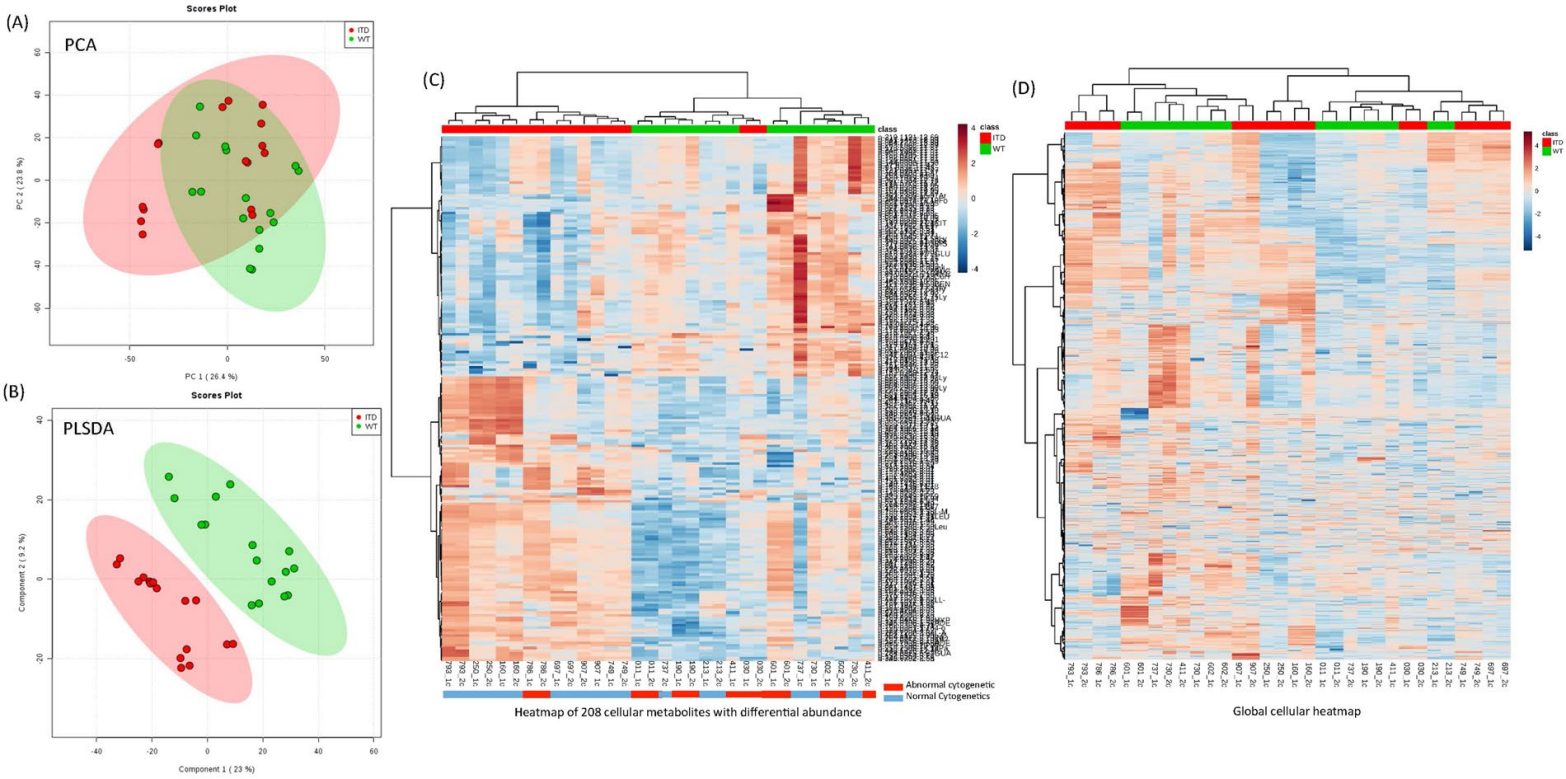
Cellular Metabolic differences between FLT3-ITD and FLT3-WT AML. (**A**) PCA plots of cellular samples. Unbiased multivariate analysis does not show clear separation of samples based on FLT3 status groups. (**B**) PLSDA plot of shows global separation of pediatric AML patients by FLT3 status (FLT3-ITD n = 8 and FLT3-WT n = 8, all samples were run in duplicate). (**C**) Heatmap shows relative abundance patterns of 208 cellular metabolites (known and un-annotated) with significantly differential abundance according to FLT3 status. Clustering within the heatmap shows a clear distinction of several metabolites between in FLT-WT and FLT3-ITD patients. (**D**) Heatmap of the global metabolome for leukemic cells from AML patients.

Multivariate analyses were conducted to evaluate the effectiveness of discriminating metabolomes according to FLT3 status. The principal component analysis (PCA) plots for an unbiased multivariate analysis did not clearly separate by FLT3 groups (Fig. 1A); however, Partial Least Square- Discriminant Analysis (PLSDA), a supervised method of multivariate analysis for discrimination in-group modeling, showed clear discrimination between FLT3-WT and FLT3-ITD (Fig. 1B). Permutation tests were conducted to identify overfitting due to supervision in the model and thresholds were set at Q2 > 0.2. The Q2 score was 0.53 in plasma metabolome model, indicating classification of two groups. Figure 1C shows heatmap of the 209 plasma metabolites (annotated as well as un-annotated) with significantly different abundance between FLT3-ITD and FLT3-WT patients (additionally, Fig. 2D shows heatmap of all the metabolites detected in plasma samples).

### Cellular Metabolic features differentiating FLT3-ITD and FLT3-WT AML

Evaluation of leukemic cells obtained at diagnosis between patients with FLT3-WT or FLT3-ITD status identified 208 (annotated and un-annotated) metabolites with differential abundance by FLT3 status in univariate analysis (p < 0.05; Supplementary Figure 2 shows the corresponding volcano plot and Supplementary Table 2 lists all the metabolites). Among the annotated metabolites within patient leukemic cell samples, as shown in Table 3, 33 were significantly different between FLT3-WT and FLT3-ITD groups. 16 metabolites had lower abundance and 17 had higher abundance in FLT3-ITD patients as compared to FLT3-WT group. Less abundant metabolites included tryptophan and formyl-5-hydroxykynurenamine with a role in tryptophan metabolism pathway; sugars such as disaccharide 6 C/6 C, glucose/fructose; glyceraldehyde with role in pyruvate metabolism and gluconeogenesis; fatty acids citramalate and arachidonic acid; and organic acids such as xylene sulfonate, benzoate, glycodeoxycholic acid and succinate (which plays a role in TCA cycle). Among metabolites with higher abundance in FLT3-ITD were: amino acids (leucine, L-carnitine, L-methionine, 4-oxoproline, LL-2,6-Diaminoheptanedioate and L-acetylcarnitine), nucleosides (guanosine, adenosine, inosine, hypoxanthine, adenosine 5′-monophosphate and guanine), lipids (Palmitoleic acid and Lyso PEs), an organic acid (glycolate), and one dipeptide (L-Leucyl-L-proline).

**Table 3:** Metabolites with significantly differential abundance in leukemia cell samples from patients with FLT3-ITD vs. without FLT3-ITD.

Metabolite	Classification	Associated Pathway	p-value	FDR	Fold Change
*Xylenesulfonate*	*Organic Acids*	*N/A*	*1.09E-06*	*0.0005*	*0.8515*
*Succinate*	*Organic Acids*	*TCA Cycle; Alanine, aspartate, and glutamate metabolism; Propanoate Metabolism; Tyrosine Metabolism; Butanoate metabolism; Phenylalanine metabolism; Glyoxlyate and dicarboxylate metabolism*	*1.61E-06*	*0.0005*	*0.8708*
*Disaccharide-6C/6 C*	*Sugars*	*N/A*	*1.79E-06*	*0.0005*	*0.7703*
L-Acetylcarnitine	Amino Acids	Fatty Acid Metabolism	2.35E-06	0.0005	3.7810
*Glyceraldehyde/Lactate*	*Aldehydes*	*Glycolysis or Gluconeogenesis; Pyruvate Metabolism; Propanoate metabolism*	*3.70E-06*	*0.0007*	*0.8751*
*Glucose/Fructose*	*Sugars*	*Starch and sucrose metabolism; Galactose Metabolism; Pentose Phosphate Pathway; Amino sugar and nucleotide sugar metabolism*	*5.37E-06*	*0.0008*	*0.8398*
Inosine	Nucleosides	Purine metabolism	1.87E-05	0.0018	2.2740
Adenosine 5′-Monophosphate	Nucleosides	Purine metabolism; Nitrogen metabolism	3.40E-05	0.0028	3.0967
Allopurinol	Xenobiotics	N/A	4.26E-05	0.0032	2.0377
Guanosine	Nucleosides	Purine metabolism/biosynthesis	5.29E-05	0.0037	1.9649
Hypoxanthine	Nucleosides	Purine metabolism/biosynthesis	1.27E-04	0.0060	2.7834
Adenosine	Nucleosides	Purine metabolism/biosynthesis	1.36E-04	0.0061	2.1136
LysoPE(p-526.2933–12.92; 22:6)	Lipids	Lysophospholipid Metabolism	2.18E-04	0.0084	85.5160
*Tryptophan*	*Amino Acids*	*Tryptophan Metabolism, Nitrogen metabolism, Aminoacyl-tRNA metabolism*	*6.26E-04*	*0.0184*	*0.9078*
*LysoPE(n-500.2768-12.71; 20:4)*	*Lipids*	*Lysophospholipid Metabolism*	*6.51E-04*	*0.0184*	*0.9160*
*Benzoate*	*Organic Acids*	*N/A*	*8.50E-04*	*0.0199*	*0.9075*
*Formyl-5-hydroxykynurenamine*	*Organic Acids*	*Tryptophan Metabolism*	*1.23E-03*	*0.0238*	*0.6343*
*LysoPE(n-452.2772-13.35; 16:0)*	*Lipids*	*Lysophospholipid Metabolism*	*1.28E-03*	*0.0238*	*0.9064*
*C6H12O6-HEXOSE/KETOSE/INOSITOL*	*Sugars*	*Galactose Metabolism; Amino sugar and nucleotide sugar metabolism*	*1.50E-03*	*0.0250*	*0.9202*
L-Carnitine	Amino Acids	Fatty Acid Metabolism	1.59E-03	0.0260	1.9458
LL-2,6-Diaminoheptanedioate	Amino Acids	Lysine Biosynthesis	1.70E-03	0.0268	3.2397
L-leucyl-L-proline	Dipeptides	N/A	1.77E-03	0.0274	1.6180
LysoPE(p-502.2908-12.90: 20:4)	Lipids	Lysophospholipid Metabolism	1.94E-03	0.0284	20.0570
*Arachidonic Acid (20:4)*	*Fatty Acids*	*Arachidonic acid metabolism*	*2.77E-03*	*0.0339*	*0.8431*
4-oxoproline	Amino Acids	Arginine and proline metabolism	3.63E-03	0.0382	2.1616
*Glycodeoxycholic acid*	*Organic Acids*	*N/A*	*3.94E-03*	*0.0395*	*0.9135*
Palmitoleic acid	Lipids	Fatty Acid Biosynthesis	4.07E-03	0.0400	1.6714
*Citramalate*	*Fatty Acids*	*N/A*	*4.30E-03*	*0.0417*	*0.8111*
*LysoPE(n-480.3097-14.59: 18:0)*	*Lipids*	*Lysophospholipid Metabolism*	*5.05E-03*	*0.0452*	*0.8803*
Guanine	Nucleosides	Purine metabolism/biosynthesis	5.27E-03	0.0466	3.3509
L-Methionine	Amino Acids	Aminoacyl-tRNA biosynthesis; Cysteine and methionine metabolism	5.71E-03	0.0484	2.0903
Leucine	Amino Acids	Aminoacyl-tRNA biosynthesis; valine, leucine, and isoleucine biosynthesis and degradation	5.76E-03	0.0484	1.9154
*2-alpha-D-glucosyl-D-glucose*	*sugars/lipids*	*N/A*	*5.78E-03*	*0.0484*	*0.9215*

Note: Metabolites with lower abundance in FLT3-ITD are in italics.

Similar to analysis in plasma samples, multivariate analyses were conducted to evaluate the effectiveness of discriminating cellular metabolome by FLT3 status. PCA did not differentiate FLT3 groups (Fig. 2A), but PLSDA analysis showed clear discrimination between FLT3-WT and FLT3-ITD groups (Fig. 2B). The Q2 score was 0.35 in cell metabolome model indicating clear classification of two groups. Heat-Map of 208 cellular metabolites with significantly differential abundance between FLT3-ITD and FLT3-WT patients is shown in Fig. 2C and D shows all the metabolites detected in cell samples.

We acknowledge the fact that genetic and cytogenetic lesions within these patients, specifically the higher frequency of normal cytogenetics within FLT3-ITD patients as compared to FLT3-WT, could partially contribute to the observed differences. Given the limited sample size, the sub-classification of FLT3-status by cytogenetics requires future studies in a bigger patient cohort. However, our analysis of patients with normal cytogenetics showed consistent results when analyzed by FLT3 status (63% of metabolites in plasma and 76% in cells overlapped with the whole date set, data not shown).

### Pathway analysis of metabolites identified in the FLT3-ITD vs. FLT3-WT

**Figure 3:**
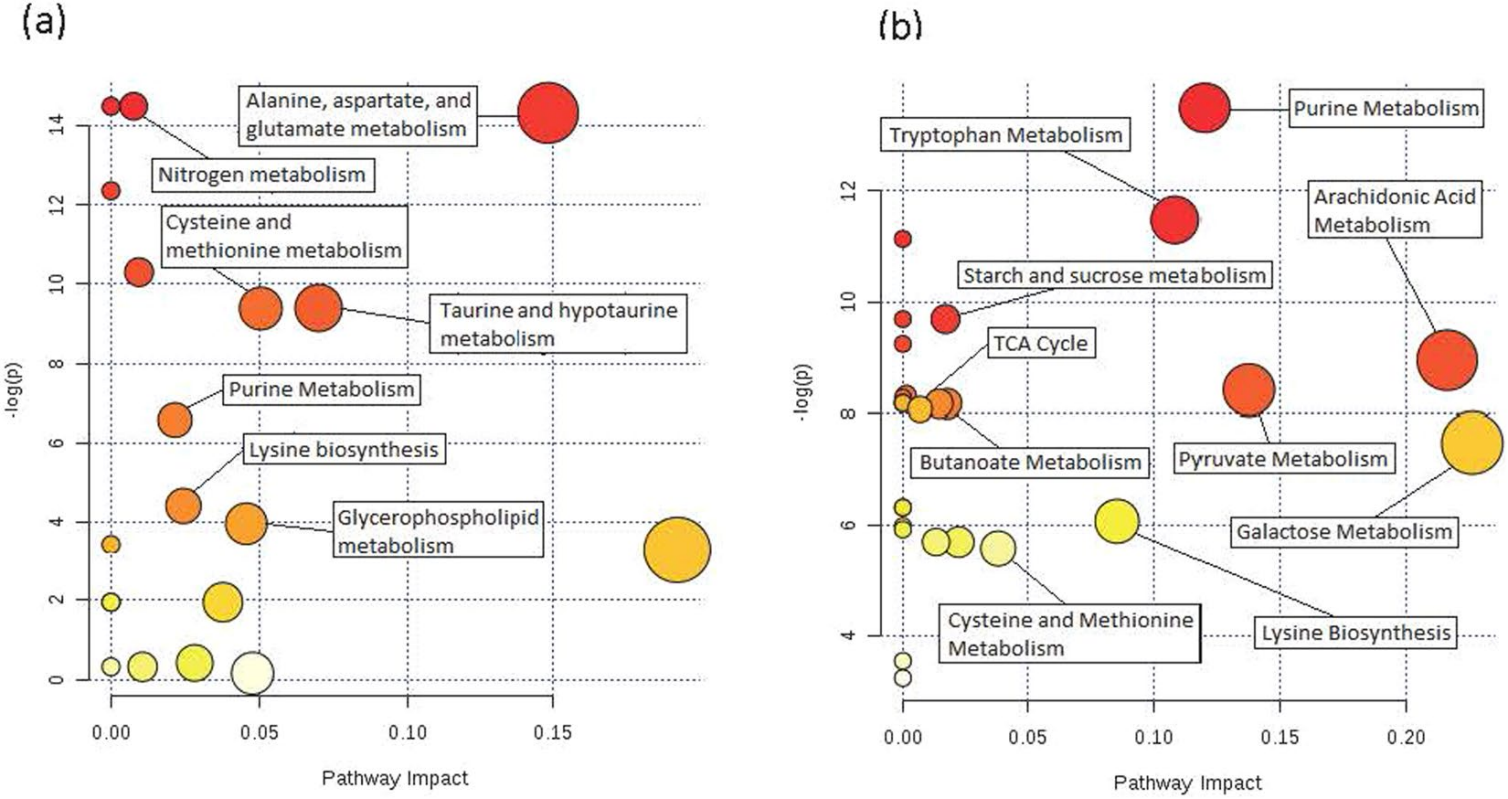
(**a**) Pathway analysis of significantly differential metabolites in plasma samples. Several of the most significantly impacted metabolic pathways have been labeled. Several impacted pathways reflected changes in the abundance of amino acids and nucleotides. (**b**) Pathway analysis of significantly differential metabolites in cell samples. The cell metabolome showed enrichment of changes to organic acid metabolic pathways. Color of circles indicate levels of statistical significance, with darker red reflecting smaller p-values and lighter colors down to white reflecting larger p-values. Circle size is meant to reflect pathway impact values as determined by pathway topology analysis. Larger circles indicate a more highly impacted metabolic pathway and smaller circles indicated a less impacted pathway.

Pathway analysis was performed using MetaboAnalyst software. The pathway library included 80 known human metabolic pathways. Significance threshold was set at FDR <0.05. Pathway analysis in the plasma metabolome identified 10 significantly impacted metabolic pathways (Fig. 3a) and included pathways such as cysteine and methionine metabolism, purine metabolism and biosynthesis, and metabolic pathways of several amino acids. Pathway analysis of the cell metabolome identified 22 significantly impacted metabolic pathways (Fig. 3b). Of interest were metabolites involved in purine metabolism or biosynthesis pathways and pyruvate metabolism that were significantly influenced by the FLT3-status. Pathways of interest associated with disease progression in both patient samples forms include purine and cysteine and methionine metabolism.

We evaluated the metabolites using KEGG database and overall the purine pathway (http://www.genome.jp/kegg-bin/show_pathway?map = map00230&show_description = show) showed a total of six metabolites with significantly different abundance between FLT3-WT and FLT3-ITD patients in both plasma and cell samples indicating differences in purine analogs levels. Further cysteine and methionine metabolism pathway (http://www.genome.jp/kegg-bin/show_pathway?map = map00270&show_description = show) were impacted in both plasma and cell samples with three metabolites S-Sulfo-L-Cysteine (Cysteine-S-Sulfate) and L-Cysteate (L-Cysteic acid) in plasma samples and L-methionine in cell samples with differential abundance. The cysteine and methionine metabolic pathway is very closely linked to several pathways that were also significantly impacted by FLT3 status, including taurine and hypotaurine metabolism; glycine, serine and threonine metabolism; and sulfur metabolism. The citrate cycle (http://www.genome.jp/kegg-bin/show_pathway?map = map00020&show_description = show) was significantly impacted in cell samples through difference in succinate abundance.

## Discussion

AML is a very heterogeneous disease with diverse clinical and biological features. One of the features associated with poor outcome is the presence of mutations, specifically an internal tandem duplication in FLT3, also known as FLT3-ITD. FLT3 has been implicated in stem cell differentiation and proliferation. Presence of FLT3-ITD with greater mutation burden (measured as higher ITD/WT allelic ration) is a well-established prognostic factor with greater allelic burden associated with significantly poor outcome. Gene expression studies to enhance understanding of FLT3 biology have identified patterns of expression differences associated with presence of FLT3-ITD. One of the newer and emerging approaches to enhance understanding of disease biology or identify biomarkers of response is metabolomics. In this study, we report the first metabolomics analysis of plasma and cell samples from pediatric AML patients with FLT3-ITD or FLT3-WT AML. Although overall FLT3-ITD status did not define the global leukemia metabolome, we identified multiple metabolic features with differential abundance between FLT3-ITD and FLT3-WT AML. While the contribution of other cytogenetic differences within FLT3-ITD and FLT3-WT cannot be ruled out, our results show multiple nucleosides and their intermediates, amino acids, organic acids, and lipids with role in cell proliferation and cancer progression to be differential in levels by FLT3 status.

Of significant interest was higher abundance of nucleosides such as guanine, hypoxanthine, inosine, adenosine, guanosine, and adenosine 5′-monophosphate within FLT3-ITD. Greater number of nucleosides within leukemic cells in FLT3-ITD as compared to FLT3-WT suggests propensity of greater cellular proliferation by providing nucleotides for DNA and RNA synthesis. Purine analogs such as ATP and GTP have been also shown to be crucial in providing cellular energy and intracellular signaling, with a role in cancer progression^19^.

Our results show that cellular metabolome of FLT3-ITD AML is less abundant in tryptophan and 5-formyl-hydroxykynurenamine. Tryptophan is converted to N-formyl kynurenine by Indolamine 2,3 dioxygenase (IDO) and to kynurenine through formamidase. IDO levels as well as kynurenine/tryptophan levels have been associated in several malignancies including AML^20,21^. Our results, although consistent with these previous findings, demonstrate the need for further in depth investigation of tryptophan metabolic pathway in context of FLT3-ITD AML. Among other amino acids, methionine was found to be more abundant in FLT3-ITD AML. L-methionine has been indicated in oncogene activation, as multiple studies have shown an increase in methionine metabolism in several cancer cell lines^22^ and patients^23^ to the extent that they are dependent on the amino acid as an energy source for cell growth. Succinate, a player in the citrate cycle, has been shown to inhibit 2-oxoglutarate dependent histone and DNA methylate enzymes, thus impacting epigenetic regulation. Given that DNA methylation is one of the hallmarks of leukemogenesis, metabolomic rewiring due to differential abundance of metabolites such as succinate or the well-established oncometabolite 2-hydroxyglutarate etc. can potentially contribute to leukemogenesis. This opens up opportunities to investigate relationships between differential succinate levels with epigenetic deregulation^24^.

L-carnitine was observed to be significantly higher in FLT3-ITD. It is involved in the transport of fatty acids across the mitochondrial membrane with the assistance of carnitine palmitoyl transferase thus allowing for cellular energy production through fatty acid β-oxidation. Serum profiling had previously shown a similar increase in carnitine abundance for patients with non-small-cell lung cancer as compared to healthy controls^25^. In addition, a recent metabolomics study reported increased carnitine levels with poor metastasis free survival outcomes in patients with different forms of soft tissue sarcoma^26^. These data suggests carnitine as a common thread among several cancer metabolomics studies, and our results further indicate that carnitine might be a useful target in future biomarker studies for FLT3-ITD pediatric AML.

Our results show lysophopholipids with differential abundance in FLT3-ITD cell samples. Lysophospholipids have been associated with disease progression in several forms of cancer, including AML, by upregulating signaling pathways^27,28^. Autotaxin, a lysophospholipid converting enzyme, in particular has been shown to have an increased activity that is associated with increased cell proliferation and migration in FLT3-ITD AML^29^ and might be potentially contributing to differential levels of LysoPEs. Overall, our results suggest differences in amino acid, purine metabolic pathways fatty acid metabolism/synthesis and various intermediates of glycolysis and TCA cycle to be enriched in FLT3-ITD vs. FLT3-WT AML.

To the best of our knowledge this report presents the first metabolomic evaluation of FLT3-ITD and FLT3-WT in pediatric AML. Our results demonstrate differential abundance of metabolites mapping to multiple pathways of biological relevance between FLT3-ITD patients as compared to FLT3-WT, these results were true for both diagnostic plasma and leukemic cell specimens. Although the global leukemia metabolome is not differentiated by FLT3 status, our results establish a precedent that certain metabolic features may differ by disease risk group categories and may have potential to be utilized as biomarkers for risk group classification. We recognize that the sample size of 16 patients is relatively small and may not adequately represent the pediatric AML patient population thus warranting a need for larger studies in pediatric AML to enhance our understanding of the biology underlying different risk group features and differential outcome. Our results open up opportunities to further enhance our understanding of FLT3 biology and ultimately identify novel drug targets that might be used to improve treatment outcome in AML.

## Materials and Methods

### Study Population

Plasma and cell samples were obtained from specimens obtained at diagnosis prior to initiation of any chemotherapy from 16 patients (8 with FLT3-ITD and 8 with FLT3-WT) enrolled in the Children’s Oncology Group-AAML1031 Clinical Trial (NCT01371981). The mononuclear cells were isolated using Ficoll-Hypaque density-gradient centrifugation. The blast percentage was >60% at the time of diagnosis and specimen collection. FLT3-ITD mutational analysis is routinely performed by Children’s Oncology Group reference labs using methods described previously^30–32^. Briefly, for mutational analysis exons 11 and 12 of FLT3 were amplified by PCR using primers 12 R, 5′-CTTTCAGCATTTTGACGGCAACC-3′, and 11 F, 5′-GCAATTTAGGTATGAAAGCCAGC-3′ followed by sequencing of bands resolved using 5% polyacrylamide gel^32^. All the patient samples with aberrant FLT3 amplification were confirmed by sequencing before post-mutational analysis for FLT3-ITD allelic ratio. FLT3-ITD +ve samples were further examined using Genescan analysis (Applied Biosystems, Foster City, CA) to determine the ratio between mutant and WT-FLT3 using previously described method^31^. Allelic ratio was calculated by dividing the peak height of the ITD product to that of the normal WT product. In cases in which more than one ITD product was present, ITD peak heights were added and divided by the WT peak height. The allelic ratio for FLT3-ITD patients was >0.80 (median allelic ratio: 1.11). AAML1031 is a randomized Phase III clinical trial that recruited newly diagnosed pediatric AML patients, aged 30 years and younger and is conducted in accordance with the Declaration of Helsinki and is registered as NCT01371981 at www.clinicaltrials.gov. The study is approved by the institutional review board (IRB) at Fred Hutchinson Cancer Center and Children’s Oncology Group-Myeloid committee and informed consent or assent has been obtained from patients or parents as appropriate. Patient characteristics are summarized in Table 1, detailed information is provided in Supplementary Table 3.

### Sample Preparation

Plasma samples were aliquoted to 100 µL volumes. 20 µL of Internal Standard Solution (Creatine-D3, L-Leucine-D10, L-Tryptophan-D3, Caffeine-D3, Leucine 13C6, L-Tyrosine 13C6, Phenylalanine 13C6, L-Tryptophan-2,3,3-D3, Aspartic Acid_t-boc, Leucine_t-boc, Propionic acid 13C3, Succinic acid_d4, Tyrosine 13C6, and Salicylic Acid-D4) and 800 µL of precipitate solution (8:1:1 acetonitrile:methanol:acetone) were added to each sample to attain a 1:8 sample to solvent ratio. Samples were vortexed and placed in 4 °C refrigerator for 30 min to precipitate proteins. Proteins were isolated and removed through centrifugation at 20,000 g for 10 min at <10 °C. 750 µL of clear supernatant were collected and placed in individual collection tubes. Supernatants were dried using nitrogen gas (N_2_ Dryer, Organomation Associates Inc., Berlin, MA). Isolated metabolites were reconstituted with addition of 100 µL of reconstitution solution (Phenylalanine_t-boc, Tryptophan_t-boc, Tyrosine_t-boc in 0.1% formic acid in water). Reconstituted metabolites were chilled on ice bath for 15 min and centrifuged using previous settings. Supernatants were collected and placed in glass liquid chromatography vials for autosampling.

Cell samples were isolated to 1 × 10^6^/mL pellets for preparation. Cell pellets were washed using 1 mL of 40 mM ammonium formate in water solution. Samples were vortexed and centrifuged at 2000 rpm for 5 min at 5 °C. Supernatant was discarded. The washing procedure was repeated twice for a total of three washes. 50 µL of 5 mM ammonium acetate in water solution was added to the cell pellets. Cell samples were then homogenized using Bead Beater (Fastprep 96, MPBio, Santa Ana, CA) at 1800 rpm for 30 sec. Samples were incubated at 4 °C for 30 min. 2 µL of internal standard solution and 1 mL of 80% methanol in water solution were added to each sample. Samples were homogenized once more using Bead Beater set to 1800 rpm for 30 sec and incubated at 4 °C for 10 min. Samples were then centrifuged at 2000 rpm for 5 min at 5 °C. 1000 µL of supernatant were collected and placed in individual collection tubes. Supernatants were dried using nitrogen gas (N_2_ Dryer, Organomation Associates Inc.). Isolated metabolites were reconstituted with addition of 30 µL of reconstitution solution. Reconstituted metabolites were chilled on ice bath for 15 min and centrifuged using previous settings. Supernatants were collected and placed in glass liquid chromatography vials for autosampling.

### Metabolomics Analysis

Global metabolomics profiling was performed on a Thermo Q-Exactive Oribtrap mass spectrometer with Thermo Ultimate 3000 UHPLC (Thermo, San Jose, CA) at Southeast Center for Integrated Metabolomics (SECIM) University of Florida. All samples were analyzed in positive and negative heated electrospray ionization with a mass resolution of 35,000 at *m/z* 200 as separate injections. Separation was achieved on an ACE 18-pfp 100 × 2.1 mm, 2 µm column with mobile phase A as 0.1% formic acid in water and mobile phase B as acetonitrile. This is a polar embedded stationary phase that provides comprehensive coverage, but does have some limitation is the coverage of very polar species. The flow rate was 350 µL/min with a column temperature of 25 °C. 4 µL was injected for negative ions and 2 µL for positive ions.

### Data Processing and Statistical Analysis

Raw data files were converted to mzxml file format using MS Convert software (ProteoWizaed 3.0). MZmine 2 was used to identify features, deisotope, align features and perform gap filling to fill in any features that may have been missed in the first alignment algorithm. All adducts and complexes were identified and removed from the data set. The data was searched against SECIM’s internal retention time metabolite library of 1100 compounds. Positive and negative mode data sets including known and unknown metabolites were merged and subjected to statistical analyses. Peak intensity values for each patient sample were grouped according to patient’s recorded FLT3 status (WT or ITD) for categorical analysis. Metabolomics statistical analysis was performed on MetaboAnalyst 3.0 web based software^17^. Datasets used for analysis were composed of merged peak area data generated by both positive and negative ionization. The metabolomics datasets included all known and unknown features detected through UHPLC-MS for analysis. Prior to analysis, MetaboAnalyst was used for data filtration and normalization functions. Missing value estimation was performed with metabolites missing over 30% of values removed. Remaining missing values were replaced by a calculated small value (half of the minimum positive value in the original data). Data filtering was performed using interquartile range. Data processing included normalization by sum, log transformation, and pareto scaling. Processed metabolomics data were then analyzed using univariate, multivariate, and clustering methods. Univariate analyses included t-test and fold change analysis. False discovery rate (FDR) was calculated to account for multiple hypothesis correction. Significance threshold was set at FDR <0.05. Multivariate analyses included principal component analysis (PCA) and partial least square discriminatory analysis (PLSDA). Clustering analysis included generation of heatmaps.

### Metabolic Pathway Enrichment and Topology Analysis

Peak intensity values for metabolites with significantly different abundance in plasma and cell samples were imported into MetaboAnalyst pathway analysis function, which generated integrated pathway enrichment and pathway topology analyses. Metabolite identifiers were converted as necessary according to synonyms listed in the human metabolome database (HMDB). The pathway impact measurement represented the sum of importance measures, generated by topology analysis, of significant metabolites normalized by importance measures of all metabolites in the associated pathway. Additionally we utilized KEGG database to map the significant metabolites on the pathways of interest^33–35^. The datasets generated during and/or analyzed during the current study are available from the corresponding author on reasonable request.

## Electronic supplementary material

Supplementary Material
