# Supplementary material for: Distinct Metabolic features differentiating FLT3-ITD AML from FLT3-WT childhood Acute Myeloid Leukemia
Source: Sci Rep. 2018 Apr 3;8:5534. doi: 10.1038/s41598-018-23863-9 (PMC5882915; doi:10.1038/s41598-018-23863-9)
Supplement: Supplementary file 1 — Supplementary Material [file 41598_2018_23863_MOESM1_ESM.docx]

**Distinct Metabolic features differentiating FLT3-ITD AML from FLT3-WT childhood Acute Myeloid Leukemia.**

Bradley Stockard^1^, Timothy Garrett^2^, Joy Guingab-Cagmat^2^, Soheil Meshinchi^3, 4^ and Jatinder Lamba^1^

**Supplementarty information:**

| Supplementary Table 1: Significant Plasma Metabolites by T-test with differential abundance between FLT3-ITD vs. FLT WT AML | | | |
| --- | --- | --- | --- |
| Metabolite ID | t.stat | p.value | FDR |
| p-219.1741-15.86 | 12.291 | 3.06E-13 | 9.00E-10 |
| p-319.1353-7.46 | 11.71 | 1.03E-12 | 1.51E-09 |
| n-166.0180-0.96 | 8.5172 | 1.67E-09 | 1.64E-06 |
| p-152.0554-1.42GUANINE | 7.7871 | 1.09E-08 | 8.01E-06 |
| p-424.3259-11.53 | 7.5883 | 1.84E-08 | 1.08E-05 |
| p-128.1071-8.18 | 6.9214 | 1.10E-07 | 4.98E-05 |
| n-158.0822-7.11 | 6.8926 | 1.19E-07 | 4.98E-05 |
| n-116.0716-7.11 | 6.8423 | 1.36E-07 | 5.00E-05 |
| p-115.0179-1.27 | 6.6043 | 2.61E-07 | 8.52E-05 |
| p-109.0760-1.85 | 6.3681 | 5.01E-07 | 0.0001471 |
| p-171.5528-7.02 | 6.2348 | 7.25E-07 | 0.00019355 |
| p-322.0758-3.23 | 6.1701 | 8.67E-07 | 0.00019778 |
| p-168.0277-1.75PYRIDINE-2,3-DICARBOXYLATE | 6.167 | 8.75E-07 | 0.00019778 |
| n-458.1064-0.95 | 6.0722 | 1.14E-06 | 0.00023914 |
| n-172.0975-8.26 | 6.0461 | 1.23E-06 | 0.00024008 |
| n-187.1083-1.23NALPHA-ACETYL-L-LYSINE | 6.0097 | 1.36E-06 | 0.00024279 |
| p-183.0861-0.73 | 5.9973 | 1.40E-06 | 0.00024279 |
| p-318.6564-13.70 | 5.9561 | 1.58E-06 | 0.00025727 |
| n-256.9911-1.01 | 5.8479 | 2.13E-06 | 0.00033003 |
| n-116.0349-1.20N-ACETYLGLYCINE | 5.8171 | 2.33E-06 | 0.00034185 |
| p-267.0588-1.40 | 5.7347 | 2.93E-06 | 0.00041033 |
| p-201.0730-6.60 | 5.6716 | 3.50E-06 | 0.00046775 |
| p-104.0707-1.034-AMINOBUTANOATE GABA | 5.648 | 3.74E-06 | 0.0004781 |
| n-74.0239-1.19 | 5.6296 | 3.94E-06 | 0.00048248 |
| p-256.2630-16.09 | 5.5624 | 4.76E-06 | 0.00055059 |
| p-110.0599-6.98 | -5.5543 | 4.87E-06 | 0.00055059 |
| p-208.0995-6.50 | -5.4671 | 6.23E-06 | 0.00067801 |
| p-211.1322-8.90 | 5.454 | 6.46E-06 | 0.00067828 |
| p-207.0430-1.30 | 5.4004 | 7.52E-06 | 0.00076189 |
| p-164.5337-11.52 | 5.3549 | 8.55E-06 | 0.00080911 |
| n-130.0506-2.18N-ACETYL-L-ALANINE | 5.3408 | 8.90E-06 | 0.00080911 |
| n-130.0872-8.24 | 5.3373 | 8.99E-06 | 0.00080911 |
| p-277.1181-9.29 | 5.3333 | 9.09E-06 | 0.00080911 |
| p-266.0106-6.15 | -5.2995 | 1.00E-05 | 0.00086408 |
| p-152.0455-1.86 | 5.2657 | 1.10E-05 | 0.0009234 |
| p-91.0539-3.28 | 5.2408 | 1.18E-05 | 0.00096319 |
| p-119.0896-1.06 | 5.1875 | 1.37E-05 | 0.0010894 |
| p-207.1023-11.38 | 5.0536 | 2.00E-05 | 0.0015357 |
| n-222.0774-7.63 | 5.0473 | 2.04E-05 | 0.0015357 |
| n-245.0926-9.23 | 5.0372 | 2.10E-05 | 0.0015406 |
| n-88.0398-2.18 | 5.0247 | 2.17E-05 | 0.0015572 |
| p-294.0145-1.79 | -4.9935 | 2.37E-05 | 0.0016263 |
| p-91.0390-1.18 | 4.9924 | 2.38E-05 | 0.0016263 |
| p-139.0865-4.30 | 4.9589 | 2.62E-05 | 0.001747 |
| p-184.0730-0.71PHOSPHOCHOLINE | 4.9427 | 2.74E-05 | 0.0017754 |
| p-300.6993-8.41 | 4.9374 | 2.78E-05 | 0.0017754 |
| n-248.1117-9.22 | 4.8871 | 3.20E-05 | 0.0020027 |
| p-170.0966-13.75Diphenylamine | 4.8513 | 3.54E-05 | 0.0021694 |
| p-185.0668-1.96 | 4.8266 | 3.80E-05 | 0.0022787 |
| n-206.0824-8.80 | 4.763 | 4.55E-05 | 0.0026713 |
| p-179.1430-10.66 | -4.7526 | 4.68E-05 | 0.0026964 |
| p-193.1420-7.44 | 4.7355 | 4.91E-05 | 0.0027753 |
| p-310.1015-11.47 | 4.7216 | 5.11E-05 | 0.0027996 |
| p-218.1211-8.57 | 4.719 | 5.15E-05 | 0.0027996 |
| p-520.3383-13.22 | 4.6969 | 5.48E-05 | 0.002925 |
| p-163.0601-0.73 | 4.6566 | 6.13E-05 | 0.0032176 |
| p-73.0648-5.92 | 4.6217 | 6.77E-05 | 0.0033602 |
| p-230.1022-10.63 | 4.6153 | 6.89E-05 | 0.0033602 |
| n-213.1494-12.10 | 4.6146 | 6.90E-05 | 0.0033602 |
| p-72.0444-0.95 | 4.6126 | 6.94E-05 | 0.0033602 |
| p-446.2589-7.99 | 4.6108 | 6.98E-05 | 0.0033602 |
| n-230.9965-7.47 | 4.6012 | 7.17E-05 | 0.003396 |
| n-256.1298-9.23 | 4.5687 | 7.85E-05 | 0.0036613 |
| p-429.2602-15.31 | 4.545 | 8.39E-05 | 0.0038516 |
| p-214.0024-1.76 | 4.536 | 8.61E-05 | 0.0038902 |
| p-372.2007-10.64 | 4.5112 | 9.22E-05 | 0.0041062 |
| p-308.2788-13.22 | 4.4897 | 9.80E-05 | 0.0042959 |
| n-447.1339-11.42 | 4.4533 | 0.0001085 | 0.0046878 |
| n-129.0556-6.733-METHYL-2-OXOVALERIC ACID | 4.4374 | 0.00011344 | 0.0048301 |
| p-118.1201-0.85 | 4.4032 | 0.0001248 | 0.0052382 |
| p-142.0617-1.20 | -4.3817 | 0.00013254 | 0.0054846 |
| p-438.2385-7.91 | 4.354 | 0.0001432 | 0.0058034 |
| p-196.1331-6.98 | 4.3515 | 0.0001442 | 0.0058034 |
| p-217.1047-6.66 | 4.329 | 0.00015351 | 0.0059338 |
| n-239.1648-12.80 | 4.3242 | 0.00015562 | 0.0059338 |
| p-161.5572-7.89 | 4.3207 | 0.00015713 | 0.0059338 |
| p-256.2993-14.59 | 4.3184 | 0.00015815 | 0.0059338 |
| n-211.1339-11.51 | 4.3131 | 0.0001605 | 0.0059338 |
| p-319.1286-8.87 | 4.3117 | 0.00016111 | 0.0059338 |
| p-166.5276-1.01 | 4.3101 | 0.00016183 | 0.0059338 |
| n-151.0069-0.98 | 4.3062 | 0.00016359 | 0.0059338 |
| n-199.0375-0.82 | 4.3017 | 0.00016566 | 0.0059354 |
| n-199.9688-0.76L-Cysteine-S-Sulfate | 4.2841 | 0.00017399 | 0.0061587 |
| p-207.0174-2.25 | 4.2776 | 0.00017715 | 0.0061796 |
| p-241.1537-6.12 | 4.2743 | 0.00017878 | 0.0061796 |
| p-160.1077-3.25 | 4.2559 | 0.00018821 | 0.0064036 |
| n-119.0349-1.07 | 4.2495 | 0.00019156 | 0.0064036 |
| p-331.6760-14.82 | -4.249 | 0.0001918 | 0.0064036 |
| p-245.0608-0.87 | 4.2232 | 0.00020606 | 0.0067996 |
| p-144.5413-2.13 | 4.2194 | 0.00020829 | 0.0067996 |
| p-189.0070-2.21 | 4.2058 | 0.00021631 | 0.0069837 |
| p-111.1165-15.10 | 4.1991 | 0.00022032 | 0.0070266 |
| p-115.1119-11.48 | 4.1912 | 0.0002252 | 0.0070266 |
| n-196.0744-0.90L-CARNITINE | 4.1884 | 0.00022697 | 0.0070266 |
| n-201.0349-0.82 | 4.188 | 0.0002272 | 0.0070266 |
| p-183.0512-1.97 | 4.1625 | 0.00024384 | 0.0074155 |
| p-143.5516-9.26 | 4.1611 | 0.00024483 | 0.0074155 |
| p-224.1278-5.97 | 4.1426 | 0.00025769 | 0.0077255 |
| n-159.0668-6.916-CARBOXYHEXANOATE | 4.1174 | 0.0002763 | 0.0081998 |
| p-130.0498-0.72 | 4.1077 | 0.00028381 | 0.0083383 |
| p-179.1065-9.99 | 4.0962 | 0.00029291 | 0.0085205 |
| n-237.1499-12.24 | 4.0897 | 0.00029823 | 0.0085354 |
| p-299.2576-15.10 | 4.0885 | 0.00029923 | 0.0085354 |
| p-204.0867-6.80 | 4.0674 | 0.00031719 | 0.0089605 |
| p-263.1960-7.14 | 4.0563 | 0.00032707 | 0.0091027 |
| n-250.0926-0.90 | 4.0517 | 0.0003312 | 0.0091027 |
| p-534.3134-8.24 | 4.0514 | 0.00033152 | 0.0091027 |
| p-274.1386-6.88 | 4.0047 | 0.00037695 | 0.010255 |
| p-172.1694-11.19 | 3.9898 | 0.00039268 | 0.010464 |
| n-160.0613-1.71 | 3.9884 | 0.00039422 | 0.010464 |
| p-137.0695-6.15 | 3.9874 | 0.00039535 | 0.010464 |
| p-194.1176-6.61 | 3.9778 | 0.00040586 | 0.010647 |
| n-225.0182-0.77 | -3.9422 | 0.00044746 | 0.011634 |
| p-376.2584-12.59 | 3.9343 | 0.00045726 | 0.011784 |
| p-288.2897-12.43 | 3.9302 | 0.00046241 | 0.011814 |
| p-190.0862-10.60METHYL INDOLE-3-ACETATE | 3.9241 | 0.00047023 | 0.01191 |
| p-196.0966-11.23 | 3.9149 | 0.00048219 | 0.012108 |
| p-135.1017-6.56 | 3.8655 | 0.00055179 | 0.013739 |
| p-279.1591-13.61 | 3.8592 | 0.00056144 | 0.013802 |
| p-154.0249-2.42 | -3.8577 | 0.00056373 | 0.013802 |
| p-262.6251-9.93 | 3.8487 | 0.00057777 | 0.013995 |
| n-260.0225-4.05 | 3.8465 | 0.00058115 | 0.013995 |
| n-539.2509-9.53 | 3.8148 | 0.00063349 | 0.015132 |
| p-221.1174-14.25 | 3.8049 | 0.00065078 | 0.015419 |
| p-203.1499-0.97 | 3.7787 | 0.0006989 | 0.016427 |
| p-144.0309-1.76 | 3.7546 | 0.00074607 | 0.017397 |
| n-188.0562-2.15N-ACETYL-DL-GLUTAMIC ACID | 3.7408 | 0.00077439 | 0.017915 |
| p-155.0466-9.35 | -3.7251 | 0.00080787 | 0.018288 |
| p-77.0382-11.48 | 3.724 | 0.00081046 | 0.018288 |
| p-213.1483-11.55 | 3.7236 | 0.00081133 | 0.018288 |
| p-153.0409-2.85 | 3.7217 | 0.00081541 | 0.018288 |
| p-212.0742-1.26 | 3.7141 | 0.00083229 | 0.018491 |
| p-170.0648-1.11 | 3.712 | 0.00083707 | 0.018491 |
| p-199.1311-8.10 | 3.701 | 0.00086217 | 0.018903 |
| n-209.0664-0.83 | 3.687 | 0.00089546 | 0.019488 |
| p-134.0242-1.90 | 3.6838 | 0.00090325 | 0.019513 |
| p-137.1323-12.17 | 3.68 | 0.00091242 | 0.019567 |
| n-143.0823-0.93 | -3.6755 | 0.00092363 | 0.019664 |
| p-231.0838-6.76 | 3.672 | 0.00093239 | 0.019708 |
| p-120.0656-0.72THREONINE/HOMOSERINE | 3.6648 | 0.00095056 | 0.019802 |
| p-113.0331-1.94 | 3.6636 | 0.00095369 | 0.019802 |
| p-226.1592-15.45 | 3.6623 | 0.00095709 | 0.019802 |
| p-287.2363-16.09 | 3.6347 | 0.0010308 | 0.021105 |
| p-245.0763-1.40 | -3.6334 | 0.0010344 | 0.021105 |
| p-213.1466-8.34 | 3.6246 | 0.0010589 | 0.021456 |
| p-930.1902-0.93 | 3.6066 | 0.0011114 | 0.022364 |
| p-160.0743-1.83 | 3.5892 | 0.0011643 | 0.023214 |
| p-168.0868-12.09 | 3.5876 | 0.0011694 | 0.023214 |
| n-213.1125-9.99 | 3.5807 | 0.001191 | 0.023485 |
| p-217.1289-1.50N-Acetyl-Arginine | 3.5579 | 0.001266 | 0.024796 |
| p-476.3077-8.01 | 3.539 | 0.0013314 | 0.025904 |
| p-155.0779-11.35 | 3.4986 | 0.0014824 | 0.028652 |
| p-357.2343-8.50 | 3.4955 | 0.0014944 | 0.028696 |
| n-131.0459-0.71ASPARAGINE | -3.4709 | 0.0015951 | 0.030212 |
| p-199.0958-9.06 | 3.4701 | 0.0015987 | 0.030212 |
| p-118.5259-1.71 | 3.4668 | 0.0016128 | 0.030212 |
| p-160.0969-1.03 | 3.4664 | 0.0016145 | 0.030212 |
| p-145.0971-1.92 | 3.4619 | 0.0016339 | 0.030383 |
| n-146.0822-0.97 | 3.4519 | 0.0016776 | 0.030999 |
| p-229.1180-1.30 | 3.4429 | 0.001718 | 0.031547 |
| p-206.0812-9.25 | 3.4358 | 0.0017505 | 0.031943 |
| p-117.5186-1.08 | 3.4322 | 0.001767 | 0.032045 |
| p-226.0705-10.05 | 3.4184 | 0.0018324 | 0.033028 |
| p-170.1095-7.65 | 3.4108 | 0.0018696 | 0.033494 |
| p-218.9950-3.99 | 3.3934 | 0.0019573 | 0.034851 |
| p-127.0868-12.74 | 3.3893 | 0.0019784 | 0.035015 |
| n-157.0506-7.27 | 3.366 | 0.0021033 | 0.036859 |
| p-247.1070-9.23 | 3.3647 | 0.0021105 | 0.036859 |
| p-213.0179-2.99 | 3.3629 | 0.0021202 | 0.036859 |
| p-524.3624-13.88 | -3.3528 | 0.0021775 | 0.037631 |
| p-197.1282-7.42 | 3.3429 | 0.0022343 | 0.038341 |
| p-168.0639-3.934-ACETAMIDOBUTANOATE | 3.3375 | 0.0022662 | 0.038341 |
| p-127.0501-6.10 | 3.3375 | 0.0022663 | 0.038341 |
| n-167.9969-0.73L-CYSTEIC ACID | -3.3368 | 0.0022707 | 0.038341 |
| p-277.1786-11.62 | 3.3335 | 0.0022899 | 0.038444 |
| p-239.1635-11.73 | 3.3246 | 0.0023438 | 0.039126 |
| p-153.0257-10.05 | 3.3221 | 0.0023596 | 0.039167 |
| p-229.1401-8.24 | 3.3132 | 0.0024146 | 0.039855 |
| p-244.1907-16.83 | -3.3039 | 0.0024744 | 0.040397 |
| p-163.0021-1.21 | 3.3038 | 0.002475 | 0.040397 |
| p-437.1924-12.83 | 3.2907 | 0.0025606 | 0.041408 |
| n-187.1338-11.373-Hydroxydecanoic acid | 3.29 | 0.0025651 | 0.041408 |
| p-118.0862-0.86BETAINE | 3.2856 | 0.0025945 | 0.041654 |
| p-112.5256-2.08 | 3.2827 | 0.0026142 | 0.041743 |
| p-128.5249-1.18 | -3.2781 | 0.0026457 | 0.042017 |
| n-215.0825-8.12 | 3.2526 | 0.0028273 | 0.044658 |
| p-208.0996-7.64 | 3.2399 | 0.0029212 | 0.045223 |
| p-120.0461-6.15 | 3.2392 | 0.0029265 | 0.045223 |
| n-291.0823-0.93 | -3.2377 | 0.0029382 | 0.045223 |
| p-133.0497-0.84 | 3.2376 | 0.002939 | 0.045223 |
| p-335.2223-8.42 | 3.2375 | 0.00294 | 0.045223 |
| n-186.0452-1.21 | 3.2344 | 0.0029637 | 0.045351 |
| n-267.1966-13.70 | 3.2295 | 0.0030008 | 0.04568 |
| p-130.0651-11.51 | 3.226 | 0.0030281 | 0.045859 |
| n-335.0731-0.92 | -3.2167 | 0.0031022 | 0.04674 |
| p-302.1361-12.74 | 3.2027 | 0.0032158 | 0.048012 |
| n-307.0778-0.91 | -3.2023 | 0.0032193 | 0.048012 |
| p-116.0475-0.97 | 3.1997 | 0.0032413 | 0.048096 |
| p-482.3244-13.17 | 3.1974 | 0.0032606 | 0.048138 |
| p-112.0395-1.24 | 3.1873 | 0.003346 | 0.049057 |
| p-205.0289-1.19 | 3.1845 | 0.0033706 | 0.049057 |
| p-236.1096-12.72 | 3.1831 | 0.0033824 | 0.049057 |
| p-172.1331-7.19 | 3.1823 | 0.0033896 | 0.049057 |
| p-270.1548-7.20 | 3.1718 | 0.0034824 | 0.049954 |
| p-152.0567-3.39 | 3.1684 | 0.0035126 | 0.049954 |
| p-282.1192-3.43 | 3.167 | 0.0035253 | 0.049954 |
| p-158.1175-1.49 | 3.166 | 0.0035345 | 0.049954 |
| p-224.1109-6.72 | 3.1655 | 0.0035391 | 0.049954 |
| p-248.1490-5.78 | 3.1639 | 0.0035535 | 0.049954 |

| Supplementary Table 2. Significant Cell Metabolites by T-test with differential abundance between FLT3-ITD vs. FLT WT AML | | | | |
| --- | --- | --- | --- | --- |
| Metabolite ID | t.stat | p.value | FDR |  |
| n-394.0818-11.45 | -7.6545 | 1.54E-08 | 2.65E-05 |  |
| n-253.1438-11.71 | -6.6107 | 2.57E-07 | 0.00021995 |  |
| n-185.0277-8.09Xylenesulfonate | -6.0869 | 1.09E-06 | 0.00050321 |  |
| n-629.2580-11.51 | -5.9729 | 1.50E-06 | 0.00050321 |  |
| n-117.0190-2.26SUCCINATE | -5.948 | 1.61E-06 | 0.00050321 |  |
| n-341.1081-0.86C12H22O11-DISACCHARIDE-6C/6C GLC-GLC/GLC-FRC/GAL-GLC | -5.9101 | 1.79E-06 | 0.00050321 |  |
| p-175.1076-1.82 | 5.8224 | 2.29E-06 | 0.00050321 |  |
| p-204.1230-3.64L-Acetylcarnitine | 5.814 | 2.35E-06 | 0.00050321 |  |
| n-89.0237-1.21GLYCERALDEHYDE/LACTATE | -5.6518 | 3.70E-06 | 0.00070554 |  |
| n-126.9048-4.31 | -5.5763 | 4.58E-06 | 0.00078555 |  |
| n-215.0321-0.72GLUCOSE/FRUCTOSE | -5.5197 | 5.37E-06 | 0.00083749 |  |
| p-263.1999-12.98 | 5.2407 | 1.18E-05 | 0.0015877 |  |
| p-317.1494-10.92 | -5.2339 | 1.20E-05 | 0.0015877 |  |
| n-201.1129-9.903 | -5.2005 | 1.32E-05 | 0.00162 |  |
| p-350.2057-11.52 | -5.1085 | 1.71E-05 | 0.00178 |  |
| n-126.9037-0.73 | 5.0858 | 1.83E-05 | 0.00178 |  |
| p-247.1287-1.81 | 5.0818 | 1.85E-05 | 0.00178 |  |
| p-269.0871-6.10INOSINE | 5.0782 | 1.87E-05 | 0.00178 |  |
| p-261.0868-10.92 | -4.982 | 2.45E-05 | 0.0021056 |  |
| n-213.0156-11.38 | -4.9814 | 2.46E-05 | 0.0021056 |  |
| p-348.0706-1.54ADENOSINE 5'-MONOPHOSPHATE | 4.8663 | 3.40E-05 | 0.002775 |  |
| p-279.6695-6.73 | 4.809 | 3.99E-05 | 0.0031128 |  |
| p-137.0456-6.10Allopurinol | 4.7861 | 4.26E-05 | 0.0031766 |  |
| p-284.0978-6.24GUANOSINE | 4.7091 | 5.29E-05 | 0.0036827 |  |
| p-531.2927-13.49 | 4.6989 | 5.45E-05 | 0.0036827 |  |
| n-119.0500-10.05 | -4.69 | 5.58E-05 | 0.0036827 |  |
| p-74.0234-11.50 | -4.6267 | 6.67E-05 | 0.0042377 |  |
| p-159.0278-1.98 | 4.5671 | 7.89E-05 | 0.0048301 |  |
| p-152.0564-6.24 | 4.5501 | 8.27E-05 | 0.0048915 |  |
| p-189.1232-1.58 | 4.537 | 8.58E-05 | 0.0049054 |  |
| p-116.1071-7.56 | -4.5043 | 9.41E-05 | 0.0052039 |  |
| p-261.1447-5.00 | 4.4816 | 0.00010022 | 0.0052638 |  |
| p-151.5440-11.52 | -4.4779 | 0.00010129 | 0.0052638 |  |
| p-284.6206-13.49 | 4.4671 | 0.00010439 | 0.0052654 |  |
| n-147.0452-11.47 | -4.4332 | 0.00011478 | 0.0056244 |  |
| p-137.0455-1.98HYPOXANTHINE | 4.398 | 0.00012665 | 0.0060337 |  |
| p-152.0359-12.74 | -4.376 | 0.00013466 | 0.0061449 |  |
| p-268.1035-5.55ADENOSINE | 4.3721 | 0.00013615 | 0.0061449 |  |
| p-189.1231-5.66 | 4.3388 | 0.00014941 | 0.0065702 |  |
| p-528.3087-13.22 | 4.2972 | 0.00016778 | 0.0071934 |  |
| p-130.0651-11.51 | -4.2453 | 0.00019381 | 0.0081068 |  |
| n-162.8389-0.71 | 4.2219 | 0.00020683 | 0.0084392 |  |
| p-526.2933-12.92LysoPE 226 | 4.2028 | 0.00021809 | 0.0084392 |  |
| n-173.0816-8.59 | -4.1979 | 0.00022104 | 0.0084392 |  |
| n-448.1726-12.46 | 4.1973 | 0.00022144 | 0.0084392 |  |
| p-297.2411-14.94 | 4.0759 | 0.00030983 | 0.011551 |  |
| n-584.2311-10.05 | -4.0387 | 0.0003433 | 0.012527 |  |
| p-144.0808-11.50 | -3.9875 | 0.00039519 | 0.01412 |  |
| p-400.7363-6.26 | 3.94 | 0.00045024 | 0.015649 |  |
| p-203.1392-3.96 | 3.9314 | 0.00046095 | 0.015649 |  |
| p-219.1121-12.69 | -3.9279 | 0.00046538 | 0.015649 |  |
| n-588.3320-13.10 | 3.9156 | 0.00048125 | 0.015872 |  |
| p-411.0932-13.95 | -3.8776 | 0.00053388 | 0.017084 |  |
| p-210.0980-6.43 | -3.8749 | 0.00053793 | 0.017084 |  |
| p-478.3212-15.80 | 3.8655 | 0.00055187 | 0.017208 |  |
| p-263.1401-7.80 | 3.8586 | 0.00056234 | 0.017222 |  |
| p-325.2263-14.46 | 3.8298 | 0.00060817 | 0.018298 |  |
| n-203.0829-7.64Tryptophan | -3.8191 | 0.00062619 | 0.018414 |  |
| p-178.1224-6.93 | -3.8061 | 0.00064867 | 0.018414 |  |
| n-500.2768-12.71LysoPE 204 | -3.8049 | 0.00065081 | 0.018414 |  |
| n-192.9451-11.37 | 3.8026 | 0.00065497 | 0.018414 |  |
| p-131.0491-11.47 | -3.7908 | 0.00067627 | 0.018707 |  |
| p-140.1435-6.01 | 3.7706 | 0.00071431 | 0.01884 |  |
| p-260.1606-6.74 | 3.7691 | 0.00071717 | 0.01884 |  |
| p-203.1020-1.23 | 3.7656 | 0.00072403 | 0.01884 |  |
| p-210.1117-6.62 | -3.7651 | 0.00072505 | 0.01884 |  |
| p-107.0488-10.05 | -3.749 | 0.00075741 | 0.019252 |  |
| p-528.3087-13.09 | 3.7461 | 0.00076333 | 0.019252 |  |
| p-323.1888-6.83 | 3.7349 | 0.00078673 | 0.019554 |  |
| p-271.1519-8.70 | 3.7265 | 0.00080496 | 0.019721 |  |
| p-203.1391-4.75 | 3.7123 | 0.00083644 | 0.019897 |  |
| n-121.0296-8.58 | -3.7064 | 0.00084975 | 0.019897 |  |
| n-206.0296-10.05 | 3.6994 | 0.00086599 | 0.019897 |  |
| p-163.1116-11.18 | 3.6991 | 0.00086671 | 0.019897 |  |
| p-530.2895-13.49 | 3.6976 | 0.00087011 | 0.019897 |  |
| p-242.5617-1.42 | 3.6894 | 0.00088964 | 0.019922 |  |
| p-194.1169-6.60 | -3.6874 | 0.00089445 | 0.019922 |  |
| p-242.2839-14.93 | -3.6577 | 0.0009689 | 0.021303 |  |
| p-219.1337-1.25 | 3.6462 | 0.00099934 | 0.021511 |  |
| n-333.0577-2.78 | 3.641 | 0.0010134 | 0.021511 |  |
| n-886.3365-10.05 | -3.6401 | 0.001016 | 0.021511 |  |
| p-143.5288-8.52 | 3.6168 | 0.0010813 | 0.022477 |  |
| p-188.0705-11.51 | -3.6146 | 0.0010878 | 0.022477 |  |
| p-140.0359-10.05 | -3.6084 | 0.0011062 | 0.022584 |  |
| p-163.1174-14.46 | 3.6032 | 0.0011214 | 0.022626 |  |
| p-208.1325-7.05 | -3.5976 | 0.0011384 | 0.022702 |  |
| n-146.0249-8.64 | 3.576 | 0.0012063 | 0.02378 |  |
| p-209.0914-11.19Formyl-5-hydroxykynurenamine | -3.5689 | 0.0012294 | 0.023796 |  |
| p-236.1677-1.38 | 3.5668 | 0.0012362 | 0.023796 |  |
| n-463.1616-10.85 | -3.563 | 0.0012488 | 0.023796 |  |
| p-107.0490-11.47 | -3.5568 | 0.0012695 | 0.023828 |  |
| n-452.2772-13.35LysoPE 160 | -3.5526 | 0.0012839 | 0.023828 |  |
| p-341.1473-12.69 | -3.5502 | 0.0012922 | 0.023828 |  |
| p-233.1489-6.75 | 3.5371 | 0.0013379 | 0.02441 |  |
| p-279.1711-8.55 | 3.5234 | 0.0013876 | 0.024547 |  |
| n-319.1296-10.08 | 3.5221 | 0.0013925 | 0.024547 |  |
| p-192.1376-7.32 | -3.5195 | 0.0014022 | 0.024547 |  |
| p-394.3527-14.92 | -3.5181 | 0.0014076 | 0.024547 |  |
| p-270.6233-12.91 | 3.5122 | 0.0014298 | 0.024547 |  |
| p-187.1075-1.99 | 3.5118 | 0.0014313 | 0.024547 |  |
| p-307.1137-8.53 | -3.5067 | 0.0014507 | 0.024633 |  |
| n-179.0556-0.73C6H12O6-HEXOSE/KETOSE/INOSITOL | -3.4949 | 0.0014971 | 0.024993 |  |
| p-180.1019-12.74 | -3.4939 | 0.001501 | 0.024993 |  |
| p-544.3404-13.06 | 3.4821 | 0.0015487 | 0.025539 |  |
| p-162.1125-0.90L-CARNITINE | 3.4716 | 0.0015925 | 0.02601 |  |
| p-150.1275-7.29 | -3.4569 | 0.0016556 | 0.026583 |  |
| p-209.1530-13.96 | -3.4562 | 0.0016585 | 0.026583 |  |
| p-191.1022-0.82LL-2,6-DIAMINOHEPTANEDIOATE | 3.4475 | 0.0016971 | 0.026777 |  |
| p-302.3057-13.88 | 3.4465 | 0.0017018 | 0.026777 |  |
| p-139.0671-0.88 | -3.4428 | 0.0017182 | 0.026789 |  |
| p-229.1538-7.30Leu Pro | 3.4306 | 0.0017747 | 0.02742 |  |
| p-261.1445-6.92 | 3.4241 | 0.0018051 | 0.02764 |  |
| p-274.6547-1.40 | 3.4169 | 0.0018401 | 0.027927 |  |
| n-283.2643-16.59 | 3.4084 | 0.0018817 | 0.028308 |  |
| p-173.5392-11.51 | -3.4032 | 0.0019073 | 0.028411 |  |
| p-161.0916-0.86 | 3.3987 | 0.0019303 | 0.028411 |  |
| p-502.2908-12.90LysoPE 204 | 3.3971 | 0.0019383 | 0.028411 |  |
| p-637.3063-16.19 | 3.3933 | 0.0019579 | 0.028456 |  |
| p-208.0969-8.80 | -3.379 | 0.0020326 | 0.02915 |  |
| p-285.1315-8.51 | -3.3769 | 0.0020442 | 0.02915 |  |
| p-302.1364-12.74 | -3.374 | 0.0020594 | 0.02915 |  |
| p-219.1251-6.43 | -3.3701 | 0.0020808 | 0.02915 |  |
| p-239.6638-15.82 | 3.3683 | 0.0020907 | 0.02915 |  |
| p-247.1289-6.70 | 3.3493 | 0.0021972 | 0.0301 |  |
| n-145.0506-7.06 | -3.349 | 0.0021991 | 0.0301 |  |
| p-224.1281-5.96 | -3.3469 | 0.0022114 | 0.0301 |  |
| p-219.1337-6.36 | 3.3379 | 0.0022642 | 0.030575 |  |
| n-194.0819-13.37 | -3.3321 | 0.0022988 | 0.030647 |  |
| p-210.4704-5.88 | 3.3283 | 0.0023213 | 0.030647 |  |
| p-520.3377-13.11 | 3.3269 | 0.0023298 | 0.030647 |  |
| p-132.4753-6.01 | 3.3246 | 0.0023441 | 0.030647 |  |
| p-503.2954-12.86 | 3.3222 | 0.0023588 | 0.030647 |  |
| p-364.2229-12.69 | -3.3167 | 0.002393 | 0.030857 |  |
| n-502.2934-13.30 | 3.306 | 0.0024604 | 0.03149 |  |
| n-190.3239-11.47 | 3.2944 | 0.0025362 | 0.032008 |  |
| p-133.1440-6.01 | 3.2941 | 0.0025383 | 0.032008 |  |
| p-104.1066-13.12 | 3.2863 | 0.00259 | 0.032422 |  |
| n-303.2330-16.07Arachidonic Acid 204 | -3.2598 | 0.0027745 | 0.033918 |  |
| p-132.8094-6.01 | 3.2587 | 0.0027826 | 0.033918 |  |
| p-504.3089-13.23 | 3.2557 | 0.0028043 | 0.033918 |  |
| n-201.1129-9.45 | 3.2533 | 0.0028217 | 0.033918 |  |
| n-325.1843-14.07 | -3.2532 | 0.0028228 | 0.033918 |  |
| p-260.1602-3.21 | 3.2524 | 0.0028282 | 0.033918 |  |
| p-432.2456-6.96 | 3.2462 | 0.0028742 | 0.034231 |  |
| p-431.3863-6.01 | 3.24 | 0.0029207 | 0.034544 |  |
| p-175.1076-2.58 | 3.2242 | 0.0030425 | 0.03551 |  |
| p-130.6022-1.42 | 3.2241 | 0.0030437 | 0.03551 |  |
| p-180.1377-6.48 | -3.2202 | 0.003074 | 0.035621 |  |
| p-157.1696-6.01 | 3.208 | 0.0031726 | 0.036299 |  |
| p-321.1435-9.23 | -3.2077 | 0.0031748 | 0.036299 |  |
| p-132.0807-11.51 | -3.2036 | 0.0032088 | 0.036444 |  |
| p-249.0863-11.51 | -3.1936 | 0.0032928 | 0.037152 |  |
| p-222.0972-0.80 | 3.1877 | 0.0033428 | 0.03747 |  |
| p-124.5915-6.55 | 3.1762 | 0.0034428 | 0.038181 |  |
| p-203.1389-6.67 | 3.1746 | 0.0034577 | 0.038181 |  |
| p-402.3567-15.47 | 3.1723 | 0.0034779 | 0.038181 |  |
| p-77.0382-11.47 | -3.17 | 0.0034981 | 0.038181 |  |
| n-144.0454-9.77 | -3.1679 | 0.0035175 | 0.038181 |  |
| p-160.0336-11.23 | -3.1644 | 0.0035495 | 0.038223 |  |
| n-190.0512-11.47 | -3.1612 | 0.0035782 | 0.038223 |  |
| n-437.1362-12.11 | 3.1578 | 0.0036098 | 0.038223 |  |
| n-128.0351-1.784-oxoproline | 3.1556 | 0.0036304 | 0.038223 |  |
| p-305.1491-11.51 | -3.154 | 0.0036446 | 0.038223 |  |
| p-165.0755-8.51 | -3.152 | 0.0036639 | 0.038223 |  |
| p-198.2092-6.01 | 3.1506 | 0.0036774 | 0.038223 |  |
| p-205.1184-2.08 | 3.145 | 0.00373 | 0.038536 |  |
| p-246.1446-6.50 | 3.1426 | 0.0037532 | 0.038543 |  |
| p-164.0703-11.47 | -3.1318 | 0.0038578 | 0.039159 |  |
| p-313.1615-8.84 | 3.1317 | 0.0038588 | 0.039159 |  |
| n-448.3075-11.46Glycodeoxycholic acid GDCA | -3.124 | 0.0039359 | 0.03948 |  |
| p-71.0855-14.93 | -3.124 | 0.0039364 | 0.03948 |  |
| p-154.0336-11.47 | -3.1203 | 0.0039729 | 0.039614 |  |
| p-301.1278-8.38 | -3.1116 | 0.0040628 | 0.040038 |  |
| p-255.2309-14.14Palmitoleic acid | 3.1105 | 0.0040743 | 0.040038 |  |
| p-350.3270-15.01 | -3.1088 | 0.0040917 | 0.040038 |  |
| p-189.1231-2.27 | 3.1072 | 0.0041089 | 0.040038 |  |
| n-147.0299-2.21CITRAMALATE | -3.0893 | 0.0042996 | 0.04166 |  |
| p-265.0814-9.23 | -3.0857 | 0.0043399 | 0.041814 |  |
| p-305.2467-15.90 | 3.0803 | 0.0044 | 0.042091 |  |
| n-681.2955-16.19 | 3.0787 | 0.0044177 | 0.042091 |  |
| p-133.1052-2.40 | 3.0759 | 0.004449 | 0.042155 |  |
| p-164.5337-11.52 | -3.0704 | 0.0045117 | 0.042158 |  |
| n-209.1545-11.71 | -3.07 | 0.0045163 | 0.042158 |  |
| n-437.1362-11.97 | -3.0682 | 0.0045371 | 0.042158 |  |
| p-178.1434-1.65 | 3.0673 | 0.0045477 | 0.042158 |  |
| p-91.0537-11.48 | -3.0557 | 0.0046833 | 0.042935 |  |
| p-170.0654-4.62 | 3.0541 | 0.004702 | 0.042935 |  |
| p-348.0702-2.56 | 3.0537 | 0.0047066 | 0.042935 |  |
| p-181.7945-3.54 | 3.0489 | 0.004764 | 0.043229 |  |
| n-129.0169-1.21 | -3.0275 | 0.0050293 | 0.045241 |  |
| n-480.3097-14.59LysoPE 180 | -3.0255 | 0.005054 | 0.045241 |  |
| n-286.1293-10.95 | 3.0247 | 0.0050649 | 0.045241 |  |
| p-217.1547-5.60 | 3.0193 | 0.0051345 | 0.045625 |  |
| p-152.0561-1.41GUANINE | 3.0087 | 0.0052727 | 0.046611 |  |
| p-241.5773-11.72 | -3.0028 | 0.0053515 | 0.047032 |  |
| n-165.0190-7.66 | 3.0011 | 0.0053751 | 0.047032 |  |
| p-339.1400-9.79 | -2.9933 | 0.0054814 | 0.047719 |  |
| p-166.1026-0.89 | 2.9882 | 0.0055527 | 0.048096 |  |
| p-233.1488-2.63 | 2.9829 | 0.0056265 | 0.04837 |  |
| n-186.0559-11.51 | -2.9807 | 0.0056579 | 0.04837 |  |
| p-150.0581-1.35L-METHIONINE | 2.9768 | 0.0057138 | 0.04837 |  |
| p-236.1279-6.83 | -2.9744 | 0.005748 | 0.04837 |  |
| p-132.1017-2.41LEUCINE | 2.9733 | 0.0057643 | 0.04837 |  |
| p-263.1028-12.69 | -2.9723 | 0.0057786 | 0.04837 |  |
| n-377.0852-0.85DISACCHARIDE 6C/6C/2-alpha-D-glucosyl-D-glucose | -2.9721 | 0.0057818 | 0.04837 |  |
| p-245.1858-8.08 | 2.9639 | 0.005901 | 0.049128 |  |
| p-221.0915-10.05 | -2.9585 | 0.0059811 | 0.049554 |  |
| p-181.4602-3.55 | 2.9553 | 0.00603 | 0.049719 |  |

| **Supplementary Table 3. Detailed Characteristic Table for Individual Study Patients** | | | | | | | | | |
| --- | --- | --- | --- | --- | --- | --- | --- | --- | --- |
| Patient | Gender | Age (Days) | Race | Ethnicity | FLT3 Status | FLT3 Allelic Ratio | NPM1 | CEBPA | Cytogenetic Test Result |
| 819011 | Female | 3814 | Unknown | Hispanic or Latino | Wild Type | 0 | Negative | Negative | Abnormal |
| 829602 | Male | 4743 | White | Not Hispanic or Latino | Wild Type | 0 | Negative | Negative | Abnormal |
| 831601 | Male | 5260 | White | Not Hispanic or Latino | Wild Type | 0 | Negative | Negative | Abnormal |
| 831411 | Female | 6551 | White | Not Hispanic or Latino | Wild Type | 0 | Negative | Negative | Abnormal |
| 826190 | Female | 7090 | White | Not Hispanic or Latino | Wild Type | 0 | Negative | Positive | Abnormal |
| 827730 | Female | 2515 | Black | Not Hispanic or Latino | Wild Type | 0 | Negative | Negative | Normal |
| 820737 | Female | 4745 | White | Not Hispanic or Latino | Wild Type | 0 | Negative | Positive | Normal |
| 822213 | Male | 5304 | White | Not Hispanic or Latino | Wild Type | 0 | Positive | Negative | Normal |
| 850793 | Male | 4589 | White | Hispanic or Latino | ITD | 0.8 | Negative | Negative | Normal |
| 842160 | Female | 4220 | Asian | Not Hispanic or Latino | ITD | 0.88 | Positive | Negative | Normal |
| 850250 | Female | 6049 | White | Not Hispanic or Latino | ITD | 0.89 | Negative | Negative | Normal |
| 851697 | Female | 1519 | White | Not Hispanic or Latino | ITD | 0.92 | Negative | Negative | Normal |
| 847907 | Female | 3452 | Asian | Not Hispanic or Latino | ITD | 1.29 | Positive | Negative | Normal |
| 848749 | Female | 4975 | White | Not Hispanic or Latino | ITD | 1.98 | Positive | Negative | Normal |
| 852030 | Male | 6494 | White | Not Hispanic or Latino | ITD | 2.94 | Negative | Negative | Abnormal |
| 857786 | Male | 4935 | White | Not Hispanic or Latino | ITD | 13.35 | Negative | Negative | Abnormal |
